# Are urological procedures in tetraplegic patients safely performed without anesthesia? a report of three cases

**DOI:** 10.1186/1754-9493-6-3

**Published:** 2012-02-20

**Authors:** Subramanian Vaidyanathan, Bakul Soni, Fahed Selmi, Gurpreet Singh, Cristian Esanu, Peter Hughes, Tun Oo, Kamesh Pulya

**Affiliations:** 1 Regional Spinal Injuries Centre, Southport and Formby District General Hospital, Town Lane, Southport PR8 6PN, UK; 2 Department of Urology, Southport and Formby District General Hospital, Town Lane, Southport PR8 6PN, UK; 3 Department of Radiology, Southport and Formby District General Hospital, Town Lane, Southport PR8 6PN, UK; 4 Department of Cardiology, Southport and Formby District General Hospital, Town lane, Southport PR8 6PN, UK

## Abstract

**Background:**

Some tetraplegic patients may wish to undergo urological procedures without anaesthesia, but these patients can develop autonomic dysreflexia if cystoscopy and vesical lithotripsy are performed without anaesthesia.

**Case presentation:**

We describe three tetraplegic patients, who developed autonomic dysreflexia when cystoscopy and laser lithotripsy were carried out without anesthesia.

In two patients, who declined anaesthesia, blood pressure increased to more than 200/110 mmHg during cystoscopy. One of these patients developed severe bleeding from bladder mucosa and lithotripsy was abandoned. Laser lithotripsy was carried out under subarachnoid block a week later in this patient, and this patient did not develop autonomic dysreflexia.

The third patient with C-3 tetraplegia had undergone correction of kyphoscoliotic deformity of spine with spinal rods and pedicular screws from the level of T-2 to S-2. Pulmonary function test revealed moderate to severe restricted curve. This patient developed vesical calculus and did not wish to have general anaesthesia because of possible need for respiratory support post-operatively. Subarachnoid block was not considered in view of previous spinal fixation. When cystoscopy and laser lithotripsy were carried out under sedation, blood pressure increased from 110/50 mmHg to 160/80 mmHg.

**Conclusion:**

These cases show that tetraplegic patients are likely to develop autonomic dysreflexia during cystoscopy and vesical lithotripsy, performed without anaesthesia. Health professionals should educate spinal cord injury patients regarding risks of autonomic dysreflexia, when urological procedures are carried out without anaesthesia. If spinal cord injury patients are made aware of potentially life-threatening complications of autonomic dysreflexia, they are less likely to decline anaesthesia for urological procedures. Subrachnoid block or epidural meperidine blocks nociceptive impulses from urinary bladder and prevents occurrence of autonomic dysreflexia. If spinal cord injury patients with lesions above T-6 decline anaesthesia, nifedipine 10 mg should be given sublingually prior to cystoscopy to prevent increase in blood pressure due to autonomic dysreflexia.

## Introduction

### Pathogenesis of autonomic dysreflexia

Autonomic dysreflexia consistently develops in patients after severe spinal cord injury above T-6 level as a result of exaggerated spinal sympathetic excitation. Spinal cord injury-induced loss of serotonergic inputs into the spinal cord intermedio-lateral cell column is proportional to the pathogenesis of autonomic dysreflexia seen after spinal cord injury. Sparing of serotonergic axons beyond a critical threshold may preserve cardiovascular regulation and prevent the development of autonomic dysreflexia [1].

### Symptoms and signs of autonomic dysreflexia

Spinal cord injury patients with lesion above T-6 may develop suddenly headache, sweating, high blood pressure, cardiac dysrhythmia, convulsions, intra-cranial bleed, and acute neurogenic pulmonary oedema as a result of autonomic dysreflexia. During an episode of autonomic dysreflexia, a significant increase in visceral sympathetic activity with coronary artery constriction can result in myocardial ischemia, even in the absence of coronary artery disease [2].

### Time of onset of autonomic dysreflexia after spinal cord injury

While autonomic dysreflexia is well recognized in the chronic stage of spinal cord injury, this potentially life-threatening complication may occur albeit rarely, in the acute phase (1 month) after spinal cord injury. The earliest episode of autonomic dysreflexia was reported on the fourth post-injury day. The trigger mechanisms for autonomic dysreflexia were somatic pain, faecal impaction, and abdominal distension. Krassioukov and associates [3] demonstrates that autonomic dysreflexia occurred in 5.7% of patients with acute spinal cord injury above T-6. Patients with severe cervical spinal cord injury are particularly susceptible to the early onset of autonomic dysreflexia.

### Autonomic dysreflexia during cystoscopy in spinal cord injury patients

Spinal cord injury patients with lesion above T-6 are susceptible to develop autonomic dysreflexia during cystoscopy. Snow and associates [4] performed cystoscopy on 102 patients with traumatic spinal cord lesion; 57 patients had sensori-motor levels above T-7, and 45 patients had levels below T-7. In 40 of the 57 patients (70%) with levels above T-7, signs and symptoms of autonomic hyper-reflexia were seen during bladder distension and cystoscopy; the remaining 17 patients (30%) did not have this response. No autonomic hyper-reflexia was observed during cystoscopy in any of the 45 patients with sensori-motor levels below T-7.

### Severe autonomic dysreflexia in spinal cord injury patients undergoing vesical lithotripsy without anaesthesia

Some tetraplegic patients may not be aware that they can develop severe autonomic dysreflexia if they undergo cystoscopy without anaesthesia. These tetraplegic patients may choose to have no anaesthesia for cystoscopy in the belief that anaesthesia can lead to chest complications and their stay in the hospital may be prolonged. We present three tetraplegic patients, in whom cystoscopy and laser lithotripsy were carried out without subarachnoid block or epidural anaesthesia. These patients developed autonomic dysreflexia during cystoscopy. One of these three patients, in whom massive increase in blood pressure was observed, developed severe bleeding from hyperaemic bladder mucosa, which led to abandoning the surgical procedure. The same patient underwent cystoscopy and lithotripsy a week later under subarachnoid block, and there was no increase in blood pressure. We discuss the importance of preventing autonomic dysreflexia in tetraplegic patients, who undergo cystoscopy and laser lithotripsy of vesical calculi.

## Case presentation

### Case 1

**Figure 1:**
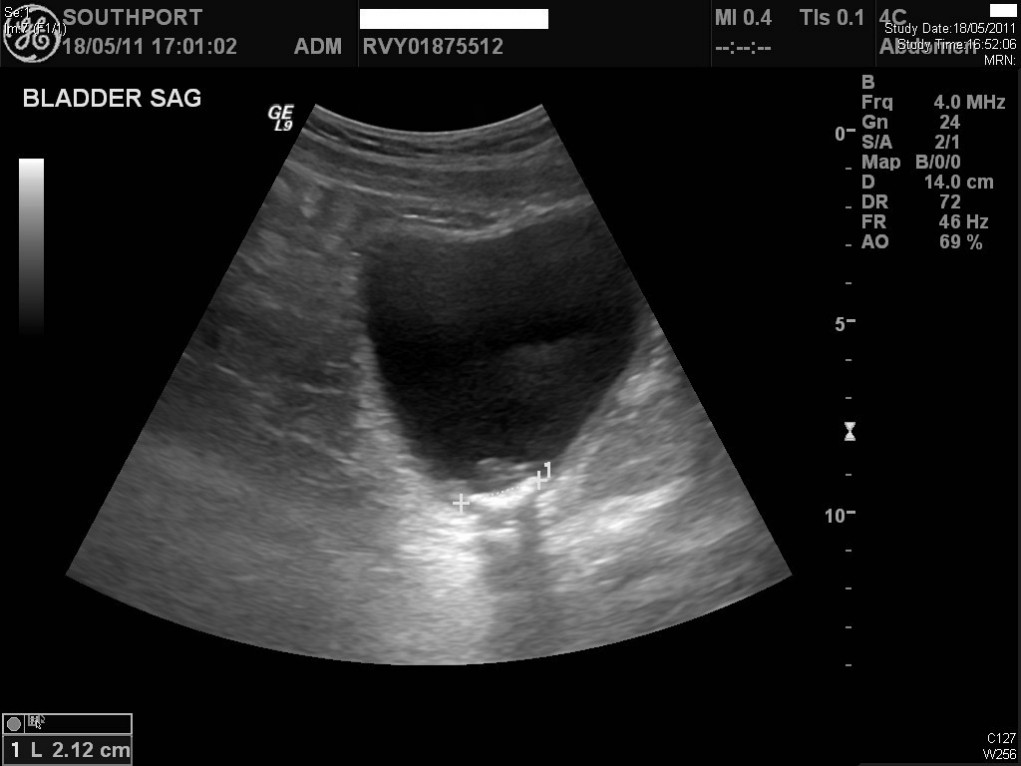
**Ultrasound examination of urinary bladder, performed on 18 May 2011, revealed multiple calculi seen posteriorly within the bladder, the largest one measuring up to 2 cm**. This patient did not develop autonomic dysreflexia, when lithotripsy was performed under subarachnoid block.

A 47-year-old-British male slipped on ice and developed C-4 complete tetraplegia (American Spinal Injury Association Grade A). Magnetic Resonance Imaging revealed C3-C4 disc prolapse with cord compression. C-3/C-4 anterior cervical discectomy, Brantigan cage and plate fusion were carried out. This patient was managing his bladder by indwelling catheter. Urethral catheter got blocked frequently. Ultrasound examination of urinary tract revealed several stones in urinary bladder (Figure 1). Cystoscopy and laser lithotripsy were carried out in June 2011. Spinal puncture was performed in right lateral position at L-3/L-4 space. Lignocaine 2% was infiltrated to skin and ligaments. Subarachnoid space was entered in first attempt with clear flow of cerebrospinal fluid. Three millilitres of 0.5% bupivacaine heavy was injected into subarachnoid space. This patient received midazolam one milligram intravenously. Teicoplanin 800 mg and amikacin one gram were also administered just before cystoscopy. Blood pressure remained stable at 100/60 mmHg during the entire operative procedure, which lasted for ninety-five minutes. There was no complication of anaesthesia or surgery.

In October 2011, this patient developed blockage of indwelling urethral catheter. Flexible cystoscopy was performed in supine posture. Special precautions were taken to avoid distension of urinary bladder during flexible cystoscopy. Flexible cystoscopy revealed recurrence of stones in urinary bladder. This patient did not manifest features of autonomic dysreflexia such as headache, sweating or goose pimples during flexible cystoscopy.

In December 2011, rigid cystoscopy and laser lithotripsy were carried out while the patient was awake and sedated. Blood pressure was 140/70 mmHg. This patient received midazolam three milligrams intravenously. Then the blood pressure was recorded as 120/70 mmHg. During cystoscopy and laser lithotripsy, blood pressure increased to 180/100 mmHg. Twenty-five milligrams of Tramadol were given intravenously three times. Blood pressure continued to rise to 210/110 mmHg. Therefore, five milligrams of Labetalol was given and this was repeated five minutes later as the blood pressure was 200/100 mmHg. This patient received Propofol 120 mg. Blood pressure decreased to 170/80 mmHg, then 110/80 mmHg, and then 90/60 mmHg. This patient developed severe bleeding from hyperaemic bladder mucosa. Therefore, laser lithotripsy was abandoned. A week later, laser lithotripsy was carried out under subarachnoid block. Initially, blood pressure was 140/70 mmHg. During later part of surgery, blood pressure remained stable at 90/50 mmHg. Laser lithotripsy was completed without any untoward incident. There was no complication of anaesthesia or surgery. The patient made an uneventful recovery.

### Case 2

**Figure 2:**
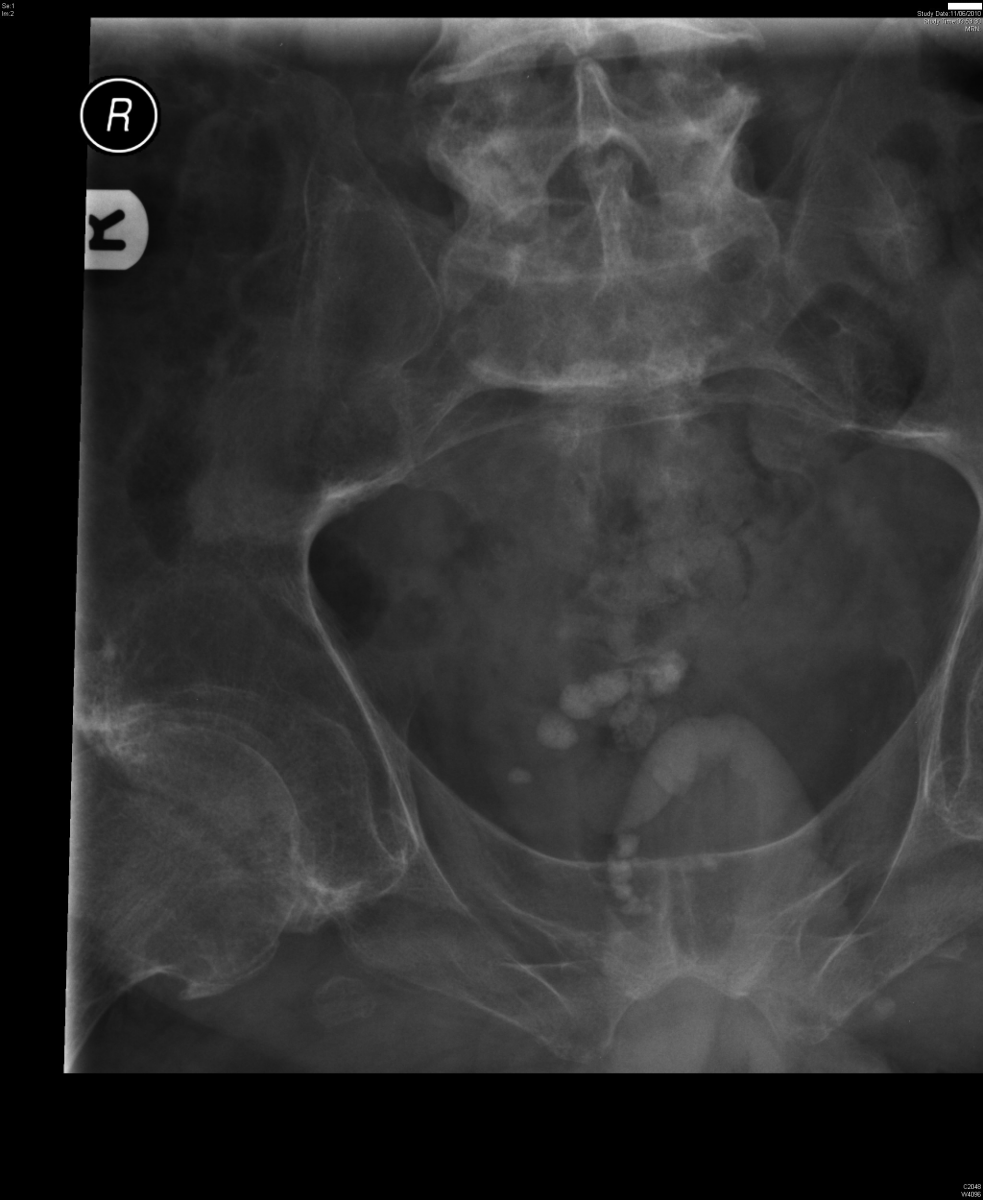
**Case number: 2. X-ray of urinary bladder, taken on 11 June 2010, showed multiple calculi in urinary bladder**. This patient did not develop autonomic dysreflexia when lithotripsy was performed under subarachnoid block.

A 49-year-old, British, male sustained complete tetraplegia at C-7 level in 1992 following a motor bike accident. This patient had been managing his bladder by intermittent catheterisation. In June 2010, this patient developed stones in urinary bladder. (Figure 2) In July 2010, cystoscopy and laser lithotripsy of vesical calculi were carried out under subarachnoid block with 3.4 ml of 0.5% bupivacaine. Systolic blood pressure remained stable around 100 mmHg during entire operative procedure, which lasted about 75 minutes. There was no immediate complication to anaesthesia or cystoscopy.

**Figure 3:**
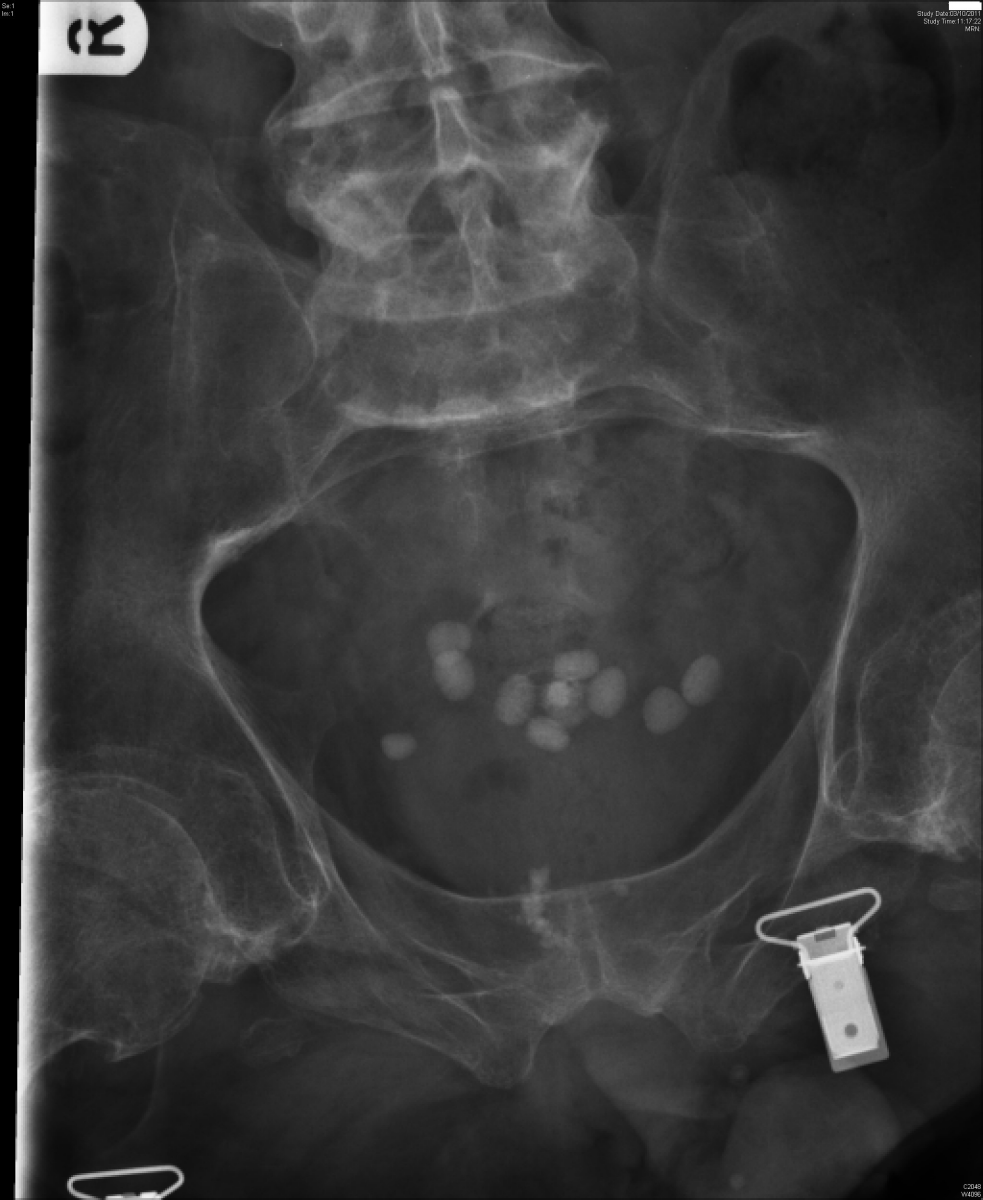
**Case number: 2. X-ray of urinary bladder, taken on 03 October 2011, showed multiple calculi within the bladder**. This patient developed severe autonomic dysreflexia when lithotripsy was carried out without anaesthesia.

In October 2011, this patient developed recurrence of stones in urinary bladder (Figure 3). This patient declined both general anaesthesia and subarachnoid block for cystoscopy and laser lithotripsy. Therefore, cystoscopy was performed without anaesthesia in December 2011. This patient received Ondansetron 4 mg and Tazocin 4.5 g prior to cystoscopy. Blood pressure increased from 150/80 mmHg to 220/120 mmHg. Two milligrams of Midazolam and 50 micrograms of Glycopyrronium bromide were administered intravenously. Blood pressure decreased to 180/90 mmHg. However, ten minutes later, blood pressure increased to 210/110 mmHg. The patient complained of headache. Ten milligrams of Labetalol were administered intravenously. Blood pressure decreased to 80/30 mmHg and then remained at 90/40 mmHg, Laser lithotripsy was completed. Apart from fall in blood pressure, no adverse effect was observed. This patient preferred to go home the same evening; he visited spinal unit three times a day for subsequent 48 hours to receive intravenous antibiotic therapy.

### Case 3

An eight-year-old, British, female sustained tetraplegia at C-3 level in a road traffic accident in 1992. Open reduction, sublaminar wiring of C-2 and C-3, and bone grafting were performed. She required tracheostomy and mechanical ventilation. She could be weaned off the ventilator and tracheostomy was closed.

**Figure 4:**
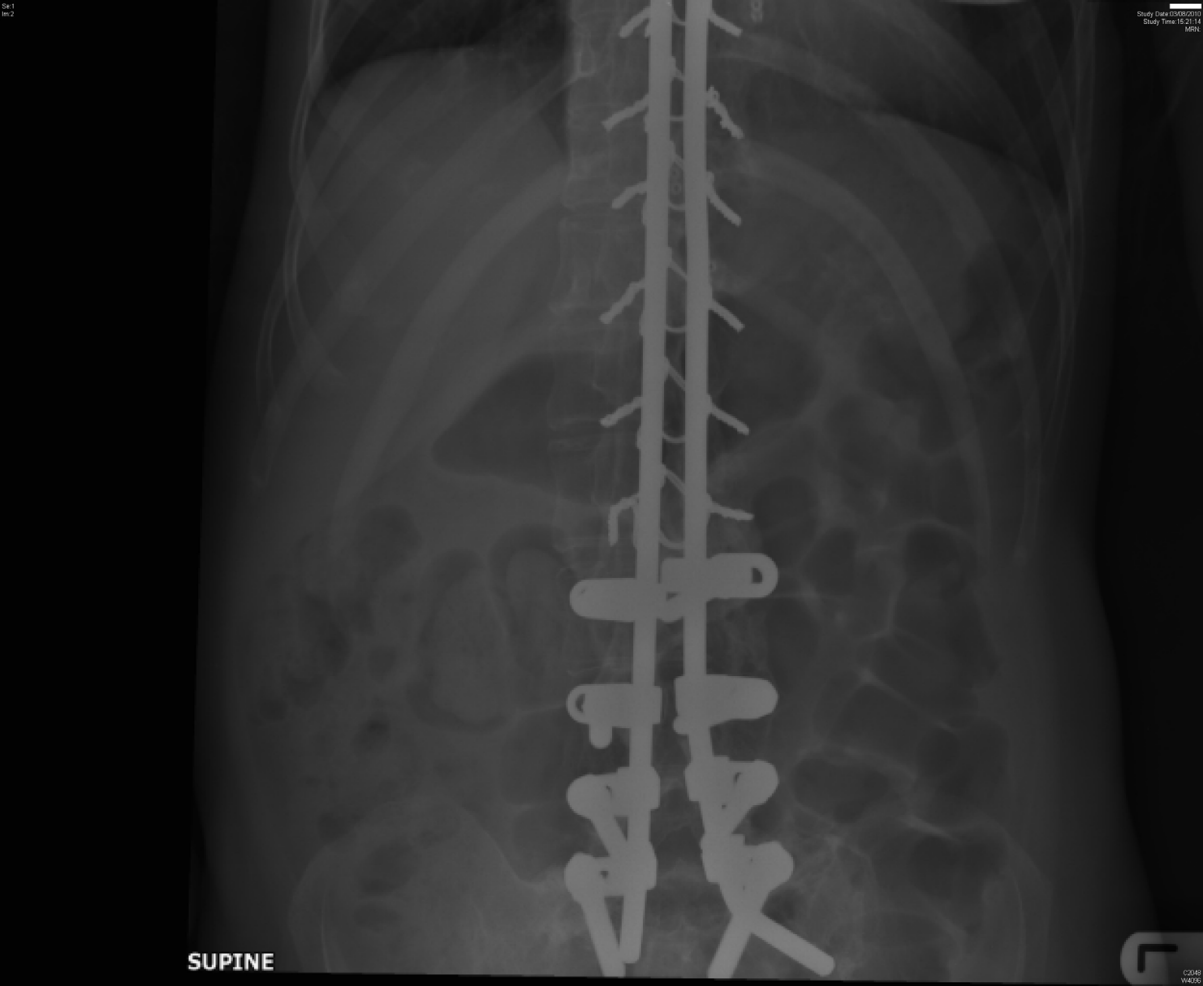
**Case number: 3. X-ray of abdomen, taken on 03 August 2010, revealed fixation with spinal rods and pedicular screws from the level of T-2 to S-2 inclusive**. Spinal fixation precluded administration of subarachnoid block.

**Figure 5:**
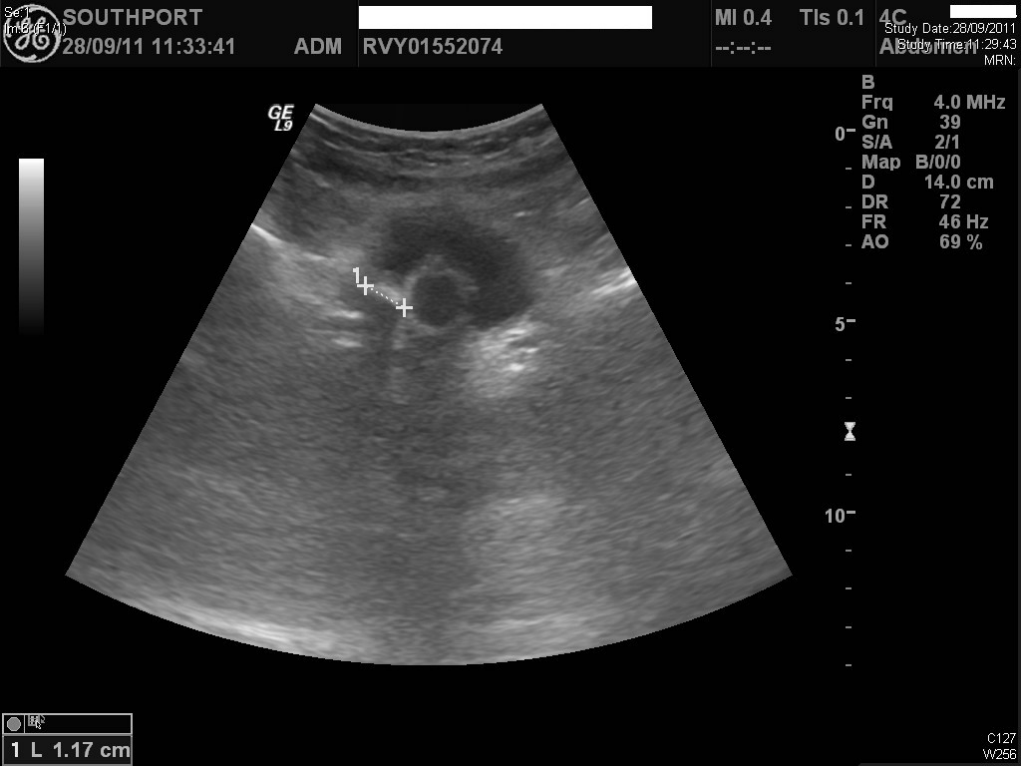
**Case number: 3. Ultrasound examination of urinary bladder, performed on 28 September 2011, revealed a 1.2 cm diameter calculus lying to the right side of the balloon of Foley catheter in the bladder**. This patient developed autonomic dysreflexia during lithotripsy, which was performed under sedation.

At the age of fifteen years, this patient underwent correction of kyphoscoliotic deformity of spine with spinal rods and pedicular screws from the level of T-2 to S-2 inclusive (Figure 4). Bone mineral density scan showed a T score of -4.8 for femoral neck. Therefore, she was prescribed Residronate 35 mg once weekly. Calcium: 2.06 mmol/L; Vitamin D-2: 0.5 ng/ml. She was advised to take AdCal D3 tablet on alternate days. She had been managing her bladder by intermittent catheterisation or by indwelling urethral catheter drainage depending upon her social engagements. Ultrasound examination of urinary tract, performed in September 2011, revealed a 1.2 cm diameter calculus lying to the right side of the balloon of Foley catheter in the bladder (Figure 5). Pulmonary function test revealed moderate to severe restricted curve. The best observations were: Peak expiratory flow was 1.88 L/second whereas predicted value was 6.44 L/second. Forced vital capacity was 0.44 L; predicted value was 3.14 L.

In view of severe restrictive lung disease and spinal fixation by rods and pedicular screws, cystoscopy and laser lithotripsy were carried out under sedation in November 2011. This patient was concerned that she might require respiratory support post-operatively if she chose to have general anaesthesia. She was rather reluctant to have general anaesthesia. Subarachnoid block was technically difficult because of spinal fixation. This patient received propofol 200 mg and midazolam two milligrams intravenously. Blood pressure was 110/50 mmHg. During cystoscopy and laser lithotripsy, blood pressure increased to 160/80 mmHg. She was given paracetamol 1 g and midazolam 1 mg intravenously. On completion of endoscopic procedure, blood pressure decreased to 110/60 mmHg.

## Discussion

### Learning point: subarachnoid block is effective in preventing autonomic dysreflexia during cystoscopy

These three cases show that spinal cord injury patients with lesion above T-6 are susceptible to develop autonomic dysreflexia during cystoscopy. Dysreflexic episodes did not occur when cystoscopy and laser lithotripsy were carried out after administering subarachnoid block in Cases 1 and 2. Whereas systolic blood pressure increased to more than 200 mmHg when cystoscopy was performed without anaesthesia, systolic blood pressure remained around 100 mmHg in the same patients while cystoscopy was carried out after they had received subarachnoid block. Thus these tetraplegic patients (cases 1 and 2) acted as their own control and proved that subarachnoid block was effective in preventing autonomic dysreflexia during cystoscopy.

"Take-home-message for the lay reader in non-medical terminology" is summarised below:

• Three persons with cervical spinal cord injury and tetraplegia developed stones in urinary bladder. Cystoscopy was performed and laser was used to break the stones (vesical lithotripsy). These procedures were carried out without anaesthesia. All three patients developed autonomic dysreflexia.

• In one patient, blood pressure increased to 210/110 mmHg; there was severe bleeding and the procedure was abandoned. A week later, this patient underwent cystoscopy after receiving spinal anaesthesia. This patient did not develop autonomic dysreflexia; stones were broken and removed from the urinary bladder without any problem.

• **We learn from these cases that tetraplegic patients are likely to develop autonomic dysreflexia if cystoscopy and removal of stones from urinary bladder are performed without anaesthesia. Spinal anaesthesia is effective in preventing autonomic dysreflexia during cystoscopy and removal of stones from urinary bladder**.

• Health professionals should discuss with spinal cord injury patients the risks of autonomic dysreflexia and possible life-threatening complications, if cystoscopy and vesical lithotripsy are carried out without anaesthesia.

### Do spinal injury patients develop autonomic dysreflexia while undergoing surgery under general anaesthesia?

General anaesthesia for a patient with prolonged spinal cord damage can be difficult because of autonomic dysreflexia, muscle wasting, and potassium changes with depolarizing muscle relaxants [5]. Mizuno and Sugimoto [6] administered general anesthesia to a 43-year-old male patient with complete sensory and motor disturbance below the upper thoracic nerves due to chronic high spinal cord injury. This patient underwent gluteus maximus musculocutaneous flap for closure of a sacral decubitus ulcer. Severe hypertension occurred probably due to autonomic hyperreflexia during the operation.

### Treatment of autonomic dysreflexia manifested during general anaesthesia

Saito and associates [7] described a 26-year-old male patient, who developed autonomic hyperreflexia while undergoing colostomy under general anaesthesia. This patient had suffered paraplegia due to the 8th cervical spinal cord injury by a traffic accident two years before. The anaesthesia was induced and maintained with propofol, fentanyl, ketamine and vecuronium. At the beginning of the operation, blood pressure varied between 135/70 to145/80 mmHg and heart rate was 55-65 beats per minute. However, blood pressure suddenly increased to 220/120 mmHg, and heart rate decreased to 43 beats per minute when the intestinal tract procedure was started. He also developed skin eruptions on his face and shoulder. Then nicardipine 1 mg was given intravenously followed by nicardipine 2 microgram per kg per minute as infusion. Subsequently, blood pressure decreased to 140-155/70-85 mmHg and heart rate increased to 75-85 beats per minute. The operation was completed without any complications. These authors recommended nicardipine for treatment of autonomic dysreflexia in a patient with upper spinal cord injury undergoing surgery under general anaesthesia.

### How is nicardipine given?

Ready-to-Use CARDENE I.V. is administered as a slow continuous infusion at a concentration of either 0.2 mg/mL (40 mg in 200 mL) or 0.1 mg/mL (20 mg in 200 mL) [8]. With constant infusion, blood pressure begins to fall within minutes. Initial dose is 5 mg/hour, which is increased by 2.5 mg/hour every 15 minutes to a maximum of 15 mg/hour depending upon blood pressure. After response is achieved, dose should be reduced to 3 mg/hour. Close monitoring of the blood pressure is required during therapy, and lowest dose of nicardipine, which is necessary to maintain stable blood pressure, should be given.

### Sevoflurane to *prevent *autonomic dysreflexia during general anaesthesia

Sevoflurane has been used to block autonomic hyperreflexia during transurethral litholapaxy under general anaesthesia in patients with complete spinal cord injury. Yoo and associates [9] studied 28 patients with chronic, complete spinal cord injury, who were scheduled to undergo transurethral litholapaxy during general anesthesia. Nine patients without spinal cord injury served as controls post hoc. Anesthesia was induced with thiopental, and sevoflurane concentrations in 50% nitrous oxide were adjusted to maintain a Bispectral Index of 40-50. When a patient developed autonomic dysreflexia during bladder distension, the target sevoflurane concentration was maintained for at least 10 minutes, and then the procedure was resumed. In patients with spinal cord injury, systolic pressure increased by 67 +/-33 mmHg, whereas heart rate decreased by 13 +/-8 beats/min during bladder distension during the first trial. The hypertensive event was associated with increases of norepinephrine concentrations, but not of epinephrine or vasopressin concentrations. Systolic pressure, heart rate, and norepinephrine concentrations did not change significantly in the control patients. These researchers found that the end-tidal concentrations of Sevoflurane to prevent autonomic dysreflexia during transurethral litholapaxy in patients were EC50 of 3.12% and EC95 of 3.83%.

### Autonomic dysreflexia during surgery performed under local anaesthesia

When spinal cord injury patients undergo surgery under local anaesthesia, they can develop autonomic dysreflexia. Intracerebral haemorrhage is an unusual complication of autonomic hyperreflexia, which can be fatal if massive bleeding occurs with subsequent brain herniation. Yoo and associates [10] presented a case of a 45-year-old tetraplegic male who suffered left basal ganglia and thalamic haemorrhage associated with autonomic dysreflexia during surgery for pressure sore defects in the prone position under local anaesthesia. This patient continued to deteriorate neurologically and died nine days later. *Yoo and associates recommend that a preventive measure should be adopted rather than episodic treatment of autonomic dysreflexia to avoid life-threatening complications*.

### Epidural meperidine to prevent autonomic dysreflexia

Epidural administration of meperidine has been shown to control autonomic dysreflexia in patients undergoing cystoscopy. Baraka and associates [11] injected meperidine 100 mg, diluted in 10 ml saline, in the epidural space at L3-L4 level in a tetraplegic patient, who had chronic spinal cord transection at C6 level before carrying out cystoscopy and transuretheral sphincterotomy. Within ten minutes and throughout the surgical procedure, the blood pressure was stable at 125/70-140/80 mmHg. These researchers postulated that epidural meperidine produced selective blockade of the spinal opiate receptors and hence blocked the nociceptive reflexes below the level of cord transection, thus preventing autonomic dysreflexia.

### Nifedipine may be given orally before cystoscopy to control autonomic dysreflexia

In some tetraplegic patients, epidural anaesthesia or subarachnoid block may not be possible because of previous spinal fixation and bone grafting, as in the case of Patient number 3, who had undergone extensive spinal fixation (Figure 4). In such patients, nifedipine may be administered *prior *to cystoscopy in order to *prevent *rise in blood pressure due to autonomic dysreflexia. Dykstra and associates [12] used calcium channel blocker, nifedipine to control autonomic hyper-reflexia during cystoscopy in 7 patients with cervical spinal cord injuries. Ten milligrams of Nifedipine alleviated autonomic dysreflexia when given sublingually during cystoscopy and *prevented *autonomic hyper-reflexia when given orally 30 minutes before cystoscopy. No adverse drug effects were observed.

### Labetalol to control hypertension due to autonomic dysreflexia

Two of three patients in this report received Labetalol intravenously to control hypertension due to autonomic dysreflexia. Labetalol is a non-selective β-adrenergic receptor antagonist, and a post-synaptic α-adrenergic receptor antagonist [13]. The β/α ratio of antagonism are 7:1 after intravenous administration (a 3:1 ratio exists after oral administration). The drug is lipid-soluble, has a 25% bioavailability, is devoid of active metabolites, and has a half-life of approximately 5.5 hours. Labetalol decreases blood pressure with a limited effect on cardiac output and heart rate at recommended dosages. Its side effects include postural hypotension/dizziness, fatigue, headache, rashes, impotence, urinary retention, gastrointestinal problems, asthma, Raynaud's phenomenon, and heart failure. In the present report, Labetalol was given to two patients, who developed rise in blood pressure because of autonomic dysreflexia. Both patients, who received Labetalol, did not manifest any side effect.

### Take home message: spinal cord physicians should discuss with spinal injury patients the risks of autonomic dysreflexia if surgery is performed without anaesthesia or under local anaesthesia

We learn from these cases that while obtaining informed consent from spinal cord injury patients, we should discuss not only the risks associated with surgery and anaesthesia but also possible complications if surgery is performed without anaesthesia. It was likely that the first two patients might not have declined anaesthesia had they been made aware of the possibility of autonomic dysreflexia occurring during cystoscopy without anaesthesia.

## Conclusion

These cases show that tetraplegic patients are likely to develop autonomic dysreflexia during cystoscopy, performed without anaesthesia. •Health professionals should educate spinal cord injury patients regarding risks of autonomic dysreflexia and possible life-threatening complications, when cystoscopy and vesical lithotripsy are carried out without anaesthesia. •If spinal cord injury patients are made aware of potentially life-threatening complications of autonomic dysreflexia, they are less likely to decline anaesthesia for urological procedures. •Subrachnoid block or epidural meperidine blocks nociceptive impulses from urinary bladder and prevents occurrence of autonomic dysreflexia. •If spinal cord injury patients with lesions above T-6 decline anaesthesia, nifedipine 10 mg should be given sublingually prior to cystoscopy to prevent increase in blood pressure due to autonomic dysreflexia. •If a spinal cord injury patient develops autonomic dysreflexia while undergoing surgery under general anaesthesia, nicardipine may be given intravenously.

## Consent

Written informed consent was obtained from all three patients for publication of the case reports and images. A copy of the written consent is available for review by the Editor-in-Chief of this journal.

## Competing interests

The authors declare that they have no competing interests. Merseyside Spinal Injuries Association kindly agreed to pay the article processing fee for this manuscript.

## Authors' contributions

SV conceived the idea and wrote the manuscript. PH reported medical images. BMS and FS were consultants in charge of the patient. All authors read and approved the final manuscript.
